# Pharmacokinetics of caffeine self-administered in overdose in a Japanese patient admitted to hospital

**DOI:** 10.1186/s40780-021-00220-z

**Published:** 2021-10-04

**Authors:** Koichiro Adachi, Satoru Beppu, Mariko Terashima, Toshiaki Fukuda, Jun Tomizawa, Makiko Shimizu, Hiroshi Yamazaki

**Affiliations:** 1 Laboratory of Drug Metabolism and Pharmacokinetics, Showa Pharmaceutical University, 3-3165 Higashi-tamagawa Gakuen, Machida, Tokyo, 194-8543 Japan; 2 Kyoto Medical Center, Fukakusa Mukaihata-cho, Fushimi-ku, Kyoto, 612-8555 Japan; 3 Himeji Medical Center, Himeji, Hyogo 670-8520 Japan

**Keywords:** Pharmacokinetic modeling, Overdose, Serum potassium, Paraxanthine

## Abstract

**Background:**

Caffeine (0.1 g) is used as a central nervous system stimulant and as a nontoxic phenotyping probe for cytochrome P450 1A2. However, an increasing number of suicide attempts by caffeine overdose have been recently reported.

**Case presentation:**

A 25-year-old woman (body weight, 43 kg) who intentionally took an overdose of 5.9 g caffeine as a suicide attempt was emergently admitted to Kyoto Medical Center. The plasma concentrations of caffeine and its primary metabolite, *N*-demethylated paraxanthine, in the current case were 100 and 7.3 μg/mL, 81 and 9.9 μg/mL, 63 and 12 μg/mL, and 21 and 14 μg/mL, at 12, 20, 30, and 56 h after oral overdose, respectively. The observed apparent terminal elimination half-life of caffeine during days 1 and 2 of hospitalization was 27 h, which is several times longer than the reported normal value. This finding implied nonlinearity of caffeine pharmacokinetics over such a wide dose range, which could affect the accuracy of values simulated by a simplified physiologically based pharmacokinetic model founded on a normal dose of 100 mg. Low serum potassium levels (2.9 and 3.5 mM) on days 1 and 2 may have been caused by the caffeine overdose in the current case**.**

**Conclusions:**

The patient underwent infusion with bicarbonate Ringer’s solution and potassium chloride and was discharged on the third day of hospitalization despite taking a potentially lethal dose of caffeine. The virtual plasma exposures of caffeine estimated using the current simplified PBPK model were higher than the measured values. The present results based on drug monitoring data and additional pharmacokinetic predictions could serve as a useful guide in cases of caffeine overdose.

## Background

Caffeine is a commonly used central nervous system stimulant [1]. Caffeine (0.1 g) is also used as a nontoxic phenotyping probe for human cytochrome P450 1A2 [2–4]. Nonetheless, a fatal caffeine overdose in a 39-year-old man resulting from the self-administered ingestion of approximately 12 g of pure caffeine anhydrous has been reported [5]. However, preemptive hemodialysis and advanced life support maneuvers, respectively, in cases of caffeine overdoses of 50 and 40 g in 39- and 19-year-old men, respectively, were successful [6, 7]. Other recent suicide attempts involving caffeine overdose in which the patients’ severe intoxication was successfully treated have been reported [8–10]. The monitoring of plasma concentrations of caffeine over a wide dose range may be considered in clinical or emergency situations.

The drug monitoring of steady-state plasma concentrations of individual patients in the clinical setting can be supported by pharmacokinetic models and simulations. Established full physiologically based pharmacokinetic (PBPK) models [11, 12] can predict drug monitoring results in patients [13–15]. We developed simplified PBPK models [16] and applied them to cases of edoxaban overdose [17] and overdosed duloxetine along with other antipsychotic drugs including quetiapine and trazodone [18]. The additional practical use of such PBPK models to the routine measurements of drug concentrations in blood has been suggested for paramedical staffs in emergency clinical practice [17, 18].

## Case presentation

Here we describe the case of a 25-year-old woman (body weight, 43 kg) who intentionally took an overdose of 5.9 g caffeine (usual clinical dose, 0.2–0.9 g/day) as a suicide attempt and was emergently admitted to Kyoto Medical Center. The patient, who had a history of neurotic depression, may have simultaneously taken lorazepam, quetiapine, risperidone, and trazodone (dosages unknown). The clinical laboratory results for this case are shown in Table 1. The patient gave written informed consent to take part in this study and for its publication. The Ethics Committee of Kyoto Medical Center approved this study (18–018).

**Table 1:** Clinical laboratory results for a patient who had taken a single oral overdose of 5900 mg caffeine

	Day 1, at 12 h	Day 2, at 30 h	Day 3, at 56 h
Aspartate aminotransferase (U/L)	19	19	17
Alanine aminotransferase (U/L)	11	11	15
Serum creatinine (mg/dL)	0.50	0.57	0.69
Creatinine clearance (mL/min)	117	102	85
Serum potassium (mmol/L)	2.9	3.5	3.7
Serum sodium (mmol/L)	137	139	141
Serum glucose (mg/dL)	190	126	87
pH [venous blood gas]	7.43	7.43	not available
Lactic acid [venous blood gas] (mmol/L)	3.01	0.96	not available

On arrival, the patient’s awareness level, as assessed using the Glasgow Coma Scale score, was eye 3, verbal 5, and motor 6 (E3V5M6) with a breathing rate of 16 breaths/min, a body temperature of 36.6 °C, a blood pressure of 113/72 mmHg, a heart rate of 83 bpm, and a QT prolongation on electro-cardiogram with a QTc of 491 ms. Laboratory data showed hypokalemia, hyperglycemia, and hyperlacticacidemia. The patient was infused with bicarbonate Ringer’s solution and potassium chloride, but was not administered activated charcoal and did not undergo artificial dialysis. By 24 h after admission, the patient’s awareness level had improved to E4V5M6 with a reduced QTc of < 430 ms. The patient refused endoscopic examination for suspected esophageal ulcer as a result of caffeine intake; consequently, lansoprazole was administered. The patient was discharged on the third day of hospitalization after abnormal vital signs had normalized.

**Fig. 1:**
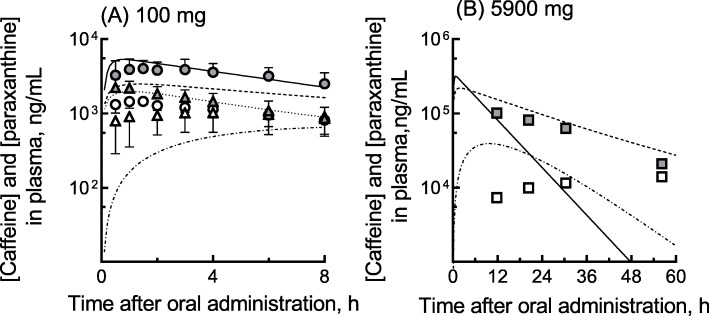
(**A**) Reported (plots) and estimated (lines) plasma concentrations of caffeine and paraxanthine in male volunteers administered single oral doses of 100 mg caffeine, and (**B**) measured (plots) and estimated (lines) concentrations of caffeine and paraxanthine in a female patient who took an oral overdose of 5900 mg caffeine. (**A**) Plasma concentrations of caffeine (closed symbols) and paraxanthine (open symbols) were taken from the literature (circles, from 4 Japanese volunteers [3]; triangles, from 30 Caucasian volunteers [4]). The plasma concentrations of caffeine (solid line) and paraxanthine (dash-dot line) estimated using the current simplified PBPK model and the estimated caffeine concentrations for virtual Japanese (dashed line) and Causation (dotted line) populations generated by the Simcyp simulator are also shown. (**B**) Measured plasma concentrations of caffeine (closed squares) and paraxanthine (open squares) in a patient who took a single oral caffeine overdose of 5900 mg. Solid and dashed lines are estimated plasma caffeine concentrations generated by the current simplified PBPK model and by the Simcyp simulator, respectively. The dash-dot line shows the PBPK-modeled paraxanthine concentrations

We measured the plasma concentrations of caffeine and its primary metabolite paraxanthine along with the other medicines and also generated PBPK-modeled concentration profiles of caffeine and its metabolite for the current patient after a self-administered single oral caffeine overdose (5.9 g); the results are shown in Fig. 1. Frozen plasma samples collected from the patient after the overdose were pharmacokinetically analyzed. After deproteinization with three volumes of methanol, the plasma concentrations of caffeine and paraxanthine were quantified by liquid chromatography using a gradient elution program followed by tandem mass spectrometry [18] according to the previously described methods [2] with slight modifications. An API4000 tandem mass analyzer (AB Sciex, Framingham, MA, USA) was used in electrospray positive ionization mode and was directly coupled to a Shimadzu LC-20 AD system equipped with an octadecylsilane (C_18_) column (XBridge, 3.5 μm, 2.1 mm × 150 mm, Waters, Milford, MA, USA). The liquid chromatography conditions for caffeine and paraxanthine were as follows: solvent A was 0.1% formic acid in water, and solvent B was 0.1% formic acid in methanol. The following gradient program was used at a flow rate of 0.20 mL/min: 0–1 min, hold at 5% B; 1.1–17 min, linear gradient from 5% B to 100% B (v/v); 17.1–21 min, hold at 100% B; and 21.1–24 min, hold at 5% B. The temperature of the column was maintained at 40 °C. Prepared samples (2.0 μL) were injected with an auto-sampler. Caffeine and paraxanthine were quantified using the *m/z* 195 → 138 and 181 → 124 transitions, respectively, with ^13^C-caffeine as an internal standard (*m/z* 198 → 140). Under these conditions, caffeine and paraxanthine levels in plasma were measurable at concentrations ≥10 ng/mL and detectable at concentrations ≥1.0 ng/mL. Inter- and intra-assay variability for caffeine and paraxanthine determinations were within 15% of coefficients of variation. Authentic caffeine and paraxanthine were purchased from Fujifilm Wako Pure Chemicals, Osaka, Japan, and ^13^C-caffeine was obtained from Sigma-Aldrich, St. Louis, MO, USA. Plasma concentrations of quetiapine, trazodone, and risperidone, which were ingested simultaneously with the caffeine, were also determined as described previously [18].

Figure 1B shows the measured plasma concentrations of caffeine and its primary metabolite paraxanthine along with the PBPK-modeled concentration profiles of the drug, which was self-administered in a single oral overdose in the current case. The plasma concentrations of caffeine and paraxanthine were 100 and 7.3 μg/mL, 81 and 9.9 μg/mL, 63 and 12 μg/mL, and 21 and 14 μg/mL at 12, 20, 30, and 56 h, respectively, after an oral overdose of 5900 mg.

Measurements of the simultaneously co-administered medicines revealed a plasma quetiapine level of 10 ng/mL 12 h after administration, with detectable (≥0.10 ng/mL) traces at 20–56 h, possibly after an approximately normal dose of 25 mg quetiapine [19]. Plasma trazodone levels of 50 and 17 ng/mL at 12 and 20 h, respectively, after administration were also determined, with detectable (≥0.10 ng/mL) traces at 30 and 56 h, possibly after an approximately normal dose of 50 mg trazodone [20], as judged by our previous simulation system [18, 21]. Similarly, detectable traces of risperidone (~ 0.10 ng/mL) were found in plasma, but the concentration could not be determined (data not shown). A rapid urine test for detecting benzodiazepines (Triage DOA, Sysmex, Kobe, Japan) showed a marginally false positive level in this case.

We also report the plasma concentration profiles for caffeine and paraxanthine generated by PBPK modeling. Based on the reported human blood concentrations of healthy volunteers who were orally treated with a normal therapeutic dose [3, 4], a simplified PBPK caffeine model consisting of gut, liver, kidney, and central compartments was set up as described previously [17, 18, 22]. The initial values for the fraction absorbed × intestinal availability (*F*_*a*_*·F*_*g*_) and hepatic clearance (*CL*_h_) were estimated from the elimination constants in empirical one-compartment models. The absorption rate constant (*k*_a_), volume of the systemic circulation (*V*_1_), and hepatic intrinsic clearance (*CL*_h,int_) values with standard deviations (as parameters for the PBPK models) were determined by fitting using nonlinear regression analyses; these final parameters are shown in Table 2. The resulting system of differential equations was solved to obtain the concentrations of caffeine and its metabolite (indicated with subscript m) for the overdose patient in the current study:

$$ \frac{dX_g}{dt}=-{k}_a\cdotp {X}_g\kern0.5em \mathrm{when}\ \mathrm{at}\kern0.5em t=0,{X}_g(0)=F\mathrm{a}\cdotp F\mathrm{g}\cdotp dose $$

$$ {V}_h\frac{dC_h}{dt}={k}_a\cdotp {X}_g-\frac{Q_h\cdotp {C}_h\cdotp {R}_b}{K_{p,h}}-{CL}_{h,\mathit{\operatorname{int}}}\cdotp \frac{C_h}{K_{p,h}}\cdotp {f}_{u,p}+{Q}_h\cdotp {C}_b $$

$$ {V}_1\frac{d{C}_b}{dt}=-\left({Q}_h+{Q}_r\right)\cdotp {C}_b+\frac{Q_h\cdotp {C}_h\cdotp {R}_b}{K_{p,h}}+\frac{Q_r\cdotp {C}_r\cdotp {R}_b}{K_{p,r}} $$

$$ {V}_r\frac{d{C}_r}{dt}={Q}_r\cdotp {C}_b-\frac{Q_r\cdotp {C}_r\cdotp {R}_b}{K_{p,r}}-{CL}_r\cdotp \frac{C_r}{K_{p,r}}\cdotp {f}_{u,p} $$

$$ {V}_{h,m}\frac{d{C}_{h,m}}{dt}={Q}_h\cdotp {C}_{b,m}-\frac{Q_h\cdotp {C}_{h,m}\cdotp {R}_{b,m}}{K_{p,h,m}}+{CL}_{h,\mathit{\operatorname{int}}}\cdotp \frac{C_h}{K_{p,h}}\cdotp {f}_{u,p}-{CL}_{h,\mathit{\operatorname{int}},m}\cdotp \frac{C_{h,m}}{K_{p,h,m}}\cdotp {f}_{u,p,m} $$

$$ {V}_{1,m}\frac{d{C}_{b,m}}{dt}=-\left({Q}_h+{Q}_r\right)\cdotp {C}_{b,m}+\frac{Q_h\cdotp {C}_{h,m}\cdotp {R}_{b,m}}{K_{p,h,m}}+\frac{Q_r\cdotp {C}_{r,m}\cdotp {R}_{b,m}}{K_{p,r,m}} $$

$$ {V}_{r,m}\frac{d{C}_{r,m}}{dt}={Q}_r\cdotp {C}_{b,m}-\frac{Q_r\cdotp {C}_{r,m}\cdotp {R}_{b,m}}{K_{p,r,m}}-{CL}_{r,m}\cdotp \frac{C_{r,m}}{K_{p,r,m}}\cdotp {f}_{u,p,m} $$

where *X*_g_, *C*_h_, *C*_r_, and *C*_b_ are the amount of compound in the gut compartment and the hepatic, renal, and blood substrate concentrations, respectively. *V*_*h*_ and *V*_r_ are the liver (1.5 L) and kidney (0.28 L) volumes, and *Q*_h_/*Q*_r_ are the blood flow rates of the systemic circulation to the hepatic/renal compartments (96.6 L/h). A full PBPK modeling simulation of caffeine was also performed with caffeine-specific physicochemical parameters using the Simcyp simulator version 20 (Certara UK, Simcyp Division, Sheffield, UK) following the modified population parameters recently described [12].

**Table 2:** Physiological, experimental, and final calculated parameters for PBPK models of caffeine and paraxanthine established in this study

Parameter	Caffeine	Paraxanthine
Model input parameters		
Molecular weight	194	180
Octanol–water partition coefficient	−0.04	−0.279
Plasma unbound fraction	0.758	0.798
Blood–plasma concentration ratio	0.822	0.798
Liver–plasma concentration ratio	0.681	0.689
Fraction absorbed × intestinal availability	1	–
Absorption rate constant, 1/h	4.94 ± 0.15 ^a^	–
Volume of systemic circulation, L	18.8 ± 0.1 ^a^	74.5 ± 0.1 ^a^
Hepatic intrinsic clearance, L/h	2.70 ± 0.05 ^a^	6.66 ± 0.08 ^a^
Hepatic clearance, L/h	2.00	5.04
Renal clearance, L/h	0.06	0.15
Estimated values ^b^		
C_max_ in plasma, ng/mL	5340 (1.3) ^c^	649 (0.45) ^c^
AUC in plasma, ng h/mL	29,300 (1.1) ^c^	3440 (0.40) ^c^
Reported levels ^d^		
C_max_ in plasma, ng/mL	4020	1450
AUC in plasma, ng h/mL	26,600	8680
Bioavailability	1	–
Urinary excretion of unchanged drug	0.03	–

The plasma unbound fraction, octanol–water partition coefficient, blood-to-plasma concentration ratio, and liver-to-plasma concentration ratio of caffeine and paraxanthine were estimated using in silico tools [23]

^a^Data are means ± standard deviations by fitting to measured concentrations

^b^Values estimated by the simplified PBPK models for a normal single oral dose of 100 mg caffeine

^c^Values in parentheses are ratios to the reported/observed values taken from the literature [3]

^d^Reported values for four Japanese male volunteers administered single oral doses of 100 mg caffeine [3]

## Discussion and conclusions

The reported plasma concentrations of caffeine and its primary metabolite paraxanthine after single oral 100-mg doses of caffeine in 4 healthy Japanese and 30 Caucasian volunteers are illustrated in Fig. 1A. The mean concentrations of caffeine in Japanese subjects appeared to be higher than those in Caucasians, whereas the levels of metabolite paraxanthine were similar in these two groups. In the current study, a simplified PBPK model for caffeine was set up based on these reported plasma concentrations of caffeine and paraxanthine in Japanese volunteers. Figure 1B shows the concentrations of caffeine and paraxanthine measured at 12, 20, 30, and 56 h after the oral overdose of 5900 mg. The measured plasma concentrations of caffeine of around 100 μg/mL in the current case appeared not to have caused hepatic impairment, as judged by the clinical laboratory results shown in Table 1. Furthermore, lines of evidence based on the plasma levels of the medicines co-administered with the caffeine implied that the doses of lorazepam, quetiapine, risperidone, and trazodone were roughly equal to the normal dose in this case.

The observed terminal elimination half-life of caffeine of 27 h (calculated from the two data points on days 1 and 2 and 16 h on day 3) after the 5900-mg overdose was several times longer than the reported normal values of 2–12 h [1]. This finding implies nonlinearity of caffeine pharmacokinetics over a wide dose range as suggested [23], thereby affecting the accuracy of the values simulated by the simplified PBPK model in the present case. As a result, the observed primary metabolite paraxanthine concentrations in plasma in the current patient were much higher from day 3 than the levels predicted by the simplified PBPK model. Low serum potassium levels on days 1 and 2 (Table 1**)** may have been caused by the caffeine overdose. It has been reported that 22 cases of fatal overdosage that resulted from the oral administration of 5.3–52 g of caffeine [1]. A rapid treatment such as gastric lavage is generally proposed to be undertaken within 60 min of ingestion [24]. However, the patient underwent infusion with bicarbonate Ringer’s solution and potassium chloride and was discharged on the third day of hospitalization.

The measured caffeine concentrations in the current overdose patient clearly indicated abnormal caffeine pharmacokinetics after taking a potentially lethal dose of caffeine. The apparent limitations of the current simplified PBPK model, which was set up based on reported plasma concentration curves for normal doses, resulted from the nonlinearity of caffeine pharmacokinetics over a wide range of drug doses; however, the in vitro–in vivo extrapolation system in the full Simcyp PBPK model was not adversely affected by this nonlinearity. These results illustrate the fact that simplified PBPK simulation systems can only be utilized appropriately if the upper dosage limit for dose–concentration linearity is known. Further model assessments and the accumulation of data and knowledge from additional overdose cases will be needed. In our previous report of overdoses of quetiapine and trazodone in combination [18], the plasma concentrations of these co-administered drugs were within their linear ranges, meaning that simplified PBPK simulation systems could be successfully applied. Such simplified PBPK models for predicting the amounts of drugs taken accidentally or intentionally can be used to facilitate the assessment of such emergently admitted cases.

In hospitals, a simulator based on simplified PBPK models may be a useful addition to the routine measurements of drug concentrations in blood. The present results, based on drug monitoring data and pharmacokinetic predictions by prepared systems in advance [25, 26], could serve as a useful guide when assessing and setting the treatments for overdose patients.

## Data Availability

All data generated or analyzed during this study are included in this published article and are also available from the corresponding author on reasonable request.

## Abbreviation

PBPK: Physiologically based pharmacokinetic
